# Critical Assessment of Implantable Drug Delivery Devices in Glaucoma Management

**DOI:** 10.1155/2013/895013

**Published:** 2013-08-26

**Authors:** Dharani Manickavasagam, Moses O. Oyewumi

**Affiliations:** Department of Pharmaceutical Sciences, College of Pharmacy, Northeast Ohio Medical University, 4209 State Route 44, Rootstown, OH 44272, USA

## Abstract

Glaucoma is a group of heterogeneous disorders involving progressive optic neuropathy that can culminate into visual impairment and irreversible blindness. Effective therapeutic interventions must address underlying vulnerability of retinal ganglion cells (RGCs) to degeneration in conjunction with correcting other associated risk factors (such as elevated intraocular pressure). However, realization of therapeutic outcomes is heavily dependent on suitable delivery system that can overcome myriads of anatomical and physiological barriers to intraocular drug delivery. Development of clinically viable sustained release systems in glaucoma is a widely recognized unmet need. In this regard, implantable delivery systems may relieve the burden of chronic drug administration while potentially ensuring high intraocular drug bioavailability. Presently there are no FDA-approved implantable drug delivery devices for glaucoma even though there are several ongoing clinical studies. The paper critically assessed the prospects of polymeric implantable delivery systems in glaucoma while identifying factors that can dictate (a) patient tolerability and acceptance, (b) drug stability and drug release profiles, (c) therapeutic efficacy, and (d) toxicity and biocompatibility. The information gathered could be useful in future research and development efforts on implantable delivery systems in glaucoma.

## 1. Introduction

The public health burdens from ocular diseases/disorders are enormous. It is estimated that about 9.1 million American adults have one of the major retinal degenerations such as diabetic retinopathy, glaucoma, and macular degeneration. 

While the annual cost of adult vision problems in the US is approximately $51.4 billion including the direct medical cost, loss of productivity and other costs to caregivers and healthcare payers [1, 2].

The realization of therapeutic outcomes in ocular diseases/disorders is dependent on the ability to effectively deliver effective doses of the drugs/therapeutic agents at site of action for the intended duration of treatment. Meanwhile, the eye is a very unique vital organ that is poorly accessible to drugs/therapeutics following systemic or local administration. It is reported that only less than 5% of topically administered drug enters the eye as a result of poor permeation and extensive drug loss do occur through various mechanisms such as lacrimation, tear dilution, and tear turnover [3, 4]. Achieving the desired therapeutic outcomes from topical drug administration may be further hampered by (a) poor patient adherence to daily medication dosing instructions; (b) difficulties in accurately administering drug to the eye; and (c) variable drug efficacy. Particularly, treatment of diseases affecting posterior segment of the eye will pose another layer of challenges because of the barriers to drug distribution to the retina either from the anterior segment or through blood circulation across the tight junctions of blood-retinal barrier (BRB) [5–7].

In general, conventional drug delivery systems like eye drops, suspensions, and ointments are associated with poor drug penetration and are less likely to be effective in treating the posterior segment diseases of the eye [8]. In most protracted ocular disease/disorder, it is desirable to limit the frequency of drug administration to ensure patient acceptance of drug delivery platforms while maximizing ocular drug bioavailability. Considering the posterior segment diseases, a logical approach to achieving high intraocular drug concentrations will be through intravitreal injections [6]. However, routine application of intravitreal injections has many drawbacks which include (i) the potential fast drug elimination from the posterior chamber which will shorten duration of drug action; (ii) repeated intravitreal injections may cause complications such as vitreous hemorrhage, retinal detachment, and ocular trauma [8–10], and (iii) the invasive nature of administration. A potential viable strategy of reducing the frequency of drug administration as well as ensuring substantial drug delivery to the posterior segment in chronic ocular diseases is implantable drug delivery systems. The general trend is that patients are less likely to embrace ocular delivery platforms with associated invasiveness the paper will critically assess the progress and challenges in the design, development, and application of polymeric ocular implants for glaucoma while offering our perspectives on the future trend. With the classification of glaucoma as a neurodegenerative disorder, effective drug delivery strategies especially to the posterior eye segments will be important in achieving the desired therapeutic outcomes. Although there are many clinically approved intravitreal delivery systems for other ocular diseases/disorders, none is currently approved for glaucoma at the time of writing.

## 2. Drug Delivery Systems in Glaucoma

Glaucoma is the leading cause of permanent blindness and visual impairment worldwide. It is widely recognized as a multifactorial and neurodegenerative disorder characterized by the progressive degeneration of the retinal ganglion cells (RGCs) that form the optic nerve [4, 10]. Elevation of intraocular pressure (IOP) is a major risk factor for onset and progression of glaucoma especially the primary open-angle glaucoma. However, treatment of IOP exclusively will not be efficient for many reasons. These are (1) there are many cases of glaucoma that do not have associated IOP elevation (low-tension glaucoma); (2) there are cases of elevated IOP that did not result in glaucoma; and (3) there are cases where progression of glaucoma cannot be controlled by management of IOP. 

It is estimated that by the year 2020, about 80 million people worldwide will be affected and close to 11 million will be bilaterally blind because of the disease [11, 12]. It is expected that there will be a 50% increase in the number of people that will be afflicted with glaucoma within the next 15 years based on projected expansion of the aging population [4, 11]. Most effective strategies of glaucoma management will require intraocular delivery system for neuroprotective agents to halt/restore the associated neurodegeneration while addressing any associated risk factors (such as elevated intraocular pressure (IOP)) [13–15]. 

### 2.1. Delivery of Neuroprotective Agents in Glaucoma

The involvement of RGCs loss and degeneration of optic nerve fibers recently gained attention in the pathophysiology of glaucoma. As such, neuroprotective therapies that delay or prevent RGC loss are considered to be beneficial to preserve vision. The broad spectrum of neuroprotective intervention could include antioxidative, anticaspase, anti-inflammatory, and antiapoptotic effects. Examples of neuroprotective agents that have been investigated to restore neuronal degeneration in glaucoma include memantine, brimonidine, and neurotrophins such as ciliary neurotropic factor and nerve growth factor [14–18]. Hare et al. studied the efficacy and safety of memantine, glutamate excitotoxicity blocker, administered in monkey glaucoma model (orally delivered) and rat (systemically delivered). The motivation for use of memantine for glaucoma treatment was based on the benefits and tolerability in dementia conditions [19, 20]. Experimental results showed reduced loss of RGCs with no adverse effects to the function of visual pathways and integrity of the retina [16]. However, a clinical study on evaluation of memantine as a neuroprotectant for glaucoma did not meet the primary endpoint [21]. We considered that the failed clinical experience with memantine underscored two main points: (a) the need for neuroprotective interventions to have a broad spectrum of action; (b) application of suitable delivery systems that could play influential roles in the therapeutic efficacy of neuroprotective agents. 

Brimonidine is a *α*_2_-adrenergic agonist approved for glaucoma treatment to control IOP. In addition to its IOP lowering properties, many studies on preclinical models have illustrated the neuroprotective effect of brimonidine in protecting neurons from damage [17, 22, 23]. A low-tension glaucoma clinical trial was conducted recently to compare the efficacy of brimonidine versus timolol in preserving visual function. Patients were randomly assigned to receive monotherapy with either topical brimonidine tartrate (0.2%) or timolol maleate (0.5%), and the visual field progression was studied. Visual field loss was preserved in patients treated with brimonidine despite similar IOP-lowering effect by the two drugs [15]. These results are consistent with brimonidine's known neuroprotective properties of enhancing RGC survival and blocking axonal degeneration [17, 24]. 

Neurotrophic factors have shown promise as potential drugs for treating neurodegenerative conditions since they are responsible for the growth and maintenance of neurons. Ji et al. evaluated the neuroprotective effect of ciliary neurotropic factor (CNTF) on RGCs in a rat glaucoma model. The investigators also studied the CNTF-mediated activation of Janus Kinase (JAK)/signal transducer and activation of transcription (JAK-STAT) signaling pathway to identify the potential correlation neuroprotection of RGCs by CNTF. While it is not known how the signaling pathway mediates the protection of RGCs, it was reported that JAK-STAT signaling plays an important role in halting apoptotic neuronal death [25, 26]. The intravitreal injection of CNTF in rat glaucoma models improved the survival rate of RGCs [27]. It was observed that phosphorylated STAT3 (pSTAT3) and endogenous CNTF concentrations were not sufficient enough to protect the damaged RGCs in hypertensive glaucomatous conditions. Thus, the injection of exogenous CNTF provided further neuroprotection by increasing pSTAT3 phosphorylation. Although there is a possibility that other signaling pathways could have been activated, experimental results published by Ji et al., substantiated the importance of CNTF as a promising therapeutic agent for glaucoma treatment.

Another neurotrophic factor that has been investigated in glaucoma treatment is the nerve growth factor (NGF). Topical administration of NGF in patients with severe retinal dysfunction showed promising improvement after 3 consecutive months of treatment [14]. The study exploited the high permeability properties of NGF when administered topically. This is the first human study reported using this neuroprotective agent, and topical application of NGF demonstrated inhibition of apoptosis of RGCs. Even after discontinuing NGF therapy, neuronal function was maintained for 3 months signifying reduced risk of vision loss in advanced glaucoma [14]. 

In general, deprivation of neurotrophins in glaucomatous optic nerve coupled with increase in vitreal glutamate concentrations have been implicated in RGCs loss. Thus, treatment paradigms that deliver neuroprotective agents to the eye will continue to hold great promise in managing glaucoma. Overall, it is not possible to draw definite conclusions on the safety and tolerability of neuroprotective agents from the studies conducted so far especially in chronic applications as required in glaucoma management. We are of the opinion that clinical viability of neuroprotective agents in glaucoma will require drug delivery systems that can achieve intraocular bioavailability while maintaining therapeutic drug levels at minimal dosing times.

### 2.2. Overview of Implantable Delivery Systems for Antiglaucoma Therapeutics

Ideal qualities for glaucoma drug delivery systems include the following:

- sustained delivery of drug (therapeutics) to the desired segment of the eye,
- ability to tailor drug delivery to the natural progression of the disease,
- achieve high ocular drug bioavailability,
- improve local drug activity while allaying concerns of systemic side effects or complications at the site of administration,
- drug administration should be noninvasive or minimally invasive without interfering with vision,
- drug delivery platforms should be safe and nontoxic while ensuring patient acceptance.

Implantable delivery systems can potentially surmount the challenge of patient nonadherence to therapy while offering localized controlled drug delivery. There are a diverse range of biocompatible implantable devices which include nondegradable and biodegradable drug pellets, bioerodible scleral plugs, films and discs, and polymeric matrices in different shapes and sizes that aid delivery of drugs to the posterior eye segment [28, 29]. These are considered as alternatives to repeated intravitreal injections with the ability to modulate drug release and extend intraocular half-life of therapeutics [30, 31]. Examples of sustained release implants in some preclinical glaucoma models are summarized in Table 1. Although there are a number of implantable delivery systems that are being studied in glaucoma management, none of the implants/formulations is currently FDA approved or marketed for treatment of this disease. Majority of the research work in this area have only been done in preclinical models. Examples of sustained release drug delivery systems specifically designed for glaucoma that are undergoing clinical development are listed in Table 2. Perhaps it would take several years before a viable sustained release delivery system (implantable device) will become commercially available with acceptable safety risk profiles.

#### 2.2.1. Biodegradable Ocular Implants

The key feature of implantable delivery systems that are fabricated from biodegradable polymers is that they do not require postapplication removal of implants after successfully delivering the loaded drugs/therapeutic agents. Also, biodegradability could connote potential biocompatibility in ocular tissues especially if the byproducts of degradation are safely eliminated. Biodegradable synthetic polymers such as polylactic acid (PLA), polyglycolic acid (PGA), and copolymer polylactic-co-glycolic acid (PLGA) are commonly studied. These polymers are well tolerated, biocompatible, and safe for clinical use with the possibility of modifying polymer degradation to occur over months to years. For instance, the degradation rate of PLGA is determined primarily by the ratio of lactide and glycolide monomers. Inclusion of high glycolide units will favor faster degradation. Other factors that will influence drug release kinetics from biodegradable implants are the molecular weight of the polymer and extent of crystallization. For instance, high crystalline nature and low degradation rates of PLGA containing high lactide units will support drug release predominantly by diffusion mechanism [44]. There are other factors that will affect polymer degradation and drug release such as mechanism of hydrolysis, erosion properties (bulk or surface erosion), sterilization process, shape, porosity, and implantation site, nature and type of drug to be loaded.

Compared to PLA and PLGA, polyanhydrides degrade at faster rates by surface erosion. Polyanhydrides are amenable to several chemical modifications that can change the erosion properties and rate of degradation [45]. Apart from the attractive biocompatibility profiles, the achievable linear mass loss during erosion with polyanhydrides could overcome some problems of burst (erratic) drug release. Similarly, polyorthoesters (POE) is a biodegradable hydrophobic polymer with linear drug release pattern controlled by gradual surface erosion [46]. Heller evaluated the residence time of POE IV after subconjunctival injection and observed good biocompatibility profiles and potential of achieving extended drug release [47]. 

A major challenge with most biodegradable systems is the difficulty of matching polymer mass loss to drug release. Erratic drug release and final burst release are common in cases with nonlinear erosion kinetics and usually characterized by a discontinuity of the matrix. There are reported cases that modification of the type and nature of monomeric units is effective in achieving and maintaining linear polymer erosion and drug release profiles [29, 48].

There are a number of representative ocular biodegradable implants in the literature. For instance, Wang and coworkers studied the therapeutic efficacy of PLGA films loaded with ethacrynic acid (ECA) implanted into the sclera of rabbit eyes. The films were well tolerated in vivo, and IOP was significantly lowered and maintained for 10 days [32]. The drug release profile was triphasic and release kinetics was highly dependent on the porosity of the films. This study demonstrated the potential benefit of PLGA films loaded with ECA for sustained delivery in glaucoma management via less invasive extraocular routes of administration. Also, recently Natu et al. showed that long-term sustained delivery was achieved using implantable disks prepared based on PCL disks loaded with dorzolamide administered through subconjunctival implantation in rabbit eyes. The disks were well tolerated in vivo and histological analysis of tissues from the target site indicated normal foreign body reaction suggesting that the implanted disks were biocompatible [33]. Effective IOP lowering effect was obtained compared to topically applied dorzolamide suggesting improved bioavailability of drug using biodegradable disks. 

Further to the aforementioned studies on biodegradable ocular implants, there are number of clinically available ocular implants that may be adapted in developing intravitreal drug delivery platforms in glaucoma. A notable example is Ozurdex (Allergan, Irvine, CA), which is a biodegradable rod-shaped dexamethasone implant approved by the FDA for intravitreal ocular implantation in the treatment of macular edema and uveitis affecting the posterior segment of the eye [49]. A recently conducted clinical trial on safety and efficacy of delivering brimonidine using Ozurdex PLGA platform [50] is a demonstration that the Ozurdex platform could be applied in intravitreal delivery of neuroprotective agents in glaucoma. 

#### 2.2.2. Nonbiodegradable Ocular Implants

Many nonbiodegradable polymers have been applied in making implants that can provide long-term, controlled release of a variety of drugs/therapeutics. These include polyvinyl alcohol (PVA), ethylene vinyl acetate (EVA), and silicone [51–53]. The major disadvantages with these systems are (1) the need for surgical procedure to remove the device from the site of implantation once the drug is completely released; (2) prolonged intraocular placement of the delivery systems could potentially trigger immune responses. In spite of the potential shortcomings, nonbiodegradable implants are less likely to produce burst drug release as compared to biodegradable ones. Ability to achieve predictable and linear drug release kinetics is desirable for prolonged drug action [46, 54, 55]. 

We are of the opinion that a number of nonbiodegradable ocular devices that are approved for intravitreal drug delivery in other ocular diseases could be adapted in glaucoma management. These include (i) Vitrasert, an intravitreal implantable reservoir system by Bausch & Lomb (Rochester, NY), approved for cytomegalovirus (CMV) retinitis. The implant is composed of PVA-EVA for delivery of ganciclovir over a period of 5 to 8 months [56, 57]; (ii) Retisert is similar in shape to Vitrasert is composed of PVA, silicone laminate, and is FDA approved for chronic noninfectious uveitis. This intravitreal surgical implant is designed to release corticosteroid fluocinolone acetonide in a sustained manner directly in the vitreous for about 2.5 years [58, 59]. (iii) Iluvien is another reservoir type implant like Vitrasert and Retisert designed to deliver fluocinolone acetonide for a duration of 36 months [30]. Due to its small size, it can be injected into the vitreous directly using a 25-gauge transconjunctival injector system, eliminating the need for an invasive procedure [59, 60]. 

With glaucoma currently classified as a neurodegenerative disorder, Neurotech's (NT-501) a nonbiodegradable implant has recently gained consideration for the delivery of protein therapeutics for up to a year to preserve vision cells. The implant encapsulates genetically engineered human retinal pigment epithelium (RPE) cells that secrete CNTF. The device is administered in the vitreous through a small incision in the sclera and secured at the implantation site by suturing through the titanium loop [61]. The semipermeable membrane allows the entry of nutrients and oxygen to the cells encapsulated within the implant. Similarly, the permeability of the membrane also allows the CNTF secreted by the human RPE cells to diffuse to the target site. Since the cells are sealed within the device, it prevents any possible foreign body reactions.

Neurotech's device was initially designed for potential treatment of retinitis pigmentosa (RP) and age-related macular degeneration (AMD). A phase 1 clinical trial in ten participants with RP demonstrated that this device was safe and well tolerated during the 6 months implantation period [62]. Also a phase 2 clinical study in 51 patients with advanced AMD slowed visual loss in 96.3% of treated patients at 12 months compared to the 75% of patients in control group [61, 63]. Following the successful use of this implant in other ocular neurodegenerative conditions, NT-501 could be the first device in delivering neurotrophic factor in human glaucomatous conditions [42].

#### 2.2.3. Injectable Formulations

Particulate drug delivery systems or injectable formulations such as microspheres, liposomes, and nanospheres/nanoparticles are other attractive alternatives used for extended drug release. The delivery platform involves entrapment of the drug within the nanocarrier matrix for subsequent intraocular delivery [64, 65]. Upon administration to the target site of the eye, the bioactive agent is released in a controlled manner by diffusion through the matrix or degradation of the polymer matrix. Also, the nanomicrocarriers once injected could act as a reservoir system for drug release for prolonged time period [66, 67]. Bertram et al. evaluated the release of timolol maleate from biodegradable microspheres composed of PLGA and PLA in vitro. Upon administration by subconjunctival injection, it was reported that drug release was sustained for more than 3 months, a time scale that could overcome the fundamental problem with patient adherence to treatment [34]. Since subconjunctival injection is less invasive than intravitreal injection, this study also demonstrated a potential route for prolonged drug delivery through penetration across the sclera. 

Another polymeric particulate delivery system that has currently been studied for ocular drug delivery is liposomes. Prabhu et al. developed and investigated liposomes of brimonidine tartrate for IOP lowering effects in glaucoma. The in vitro drug release showed constant delivery of therapeutics with linear release profile for extended time duration [68]. Also the in vivo IOP lowering effect was remarkably sustained after topical application. A potential limitation with many nanocarriers for ocular application is the possibility of vitreous clouding after intravitreal injection. A recent study of latanoprost loaded liposome injected subconjunctivally in rabbit eyes was reported, and the IOP lowering activity was compared with conventional daily administration of latanoprost eye drop [35]. Sustained delivery for about 50 days was achieved, and the liposomes were well tolerated in vivo and no adverse effect in ocular tissue was observed with subconjunctival injection. Also, the IOP lowering effect was superior to the conventional delivery of latanoprost by eye drops (as a standard of care option). The findings substantiated that local bioavailability and duration of action of latanoprost was improved with liposomal injection. 

## 3. Challenges of Implantable Ocular Drug Delivery

**Figure 1:**
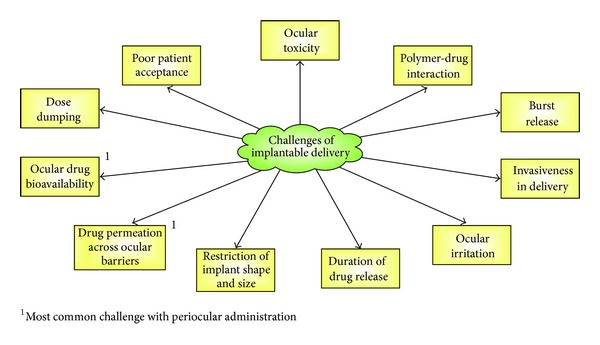
Challenges of implantable drug delivery systems in glaucoma.

The attraction with implantable drug delivery systems in ocular diseases/disorders could be attributed to many factors which include (1) intravitreal implantation would bypass the blood-retina barrier to enhance intraocular bioavailability; (2) sustained drug release will reduce the need for daily dosing which could improve patient adherence to treatment; (3) prolonged drug release will alleviate/minimize side effects associated with repeated intravitreal injection or systemic drug administration; and (4) effective drug delivery will avoid drug wastage while maximizing the efficacy of treatment. Despite the advantages of using implantable drug delivery system (DDS), there are a number of challenges as enumerated (Figure 1). 

### 3.1. Polymer-Drug Interaction

Understanding the factors that influence polymer degradation and drug release will be important in achieving sustained ocular drug release. In this regard, the type of polymer (whether homopolymer or copolymer) and the molecular weight will play substantial roles in determining hydrophilicity and the rate of degradation. For example, the hydrophilic glycolide content in PLGA is a critical parameter in determining the matrix degradation kinetics and drug release rate. PLGA 50 : 50 (PLA : PGA) exhibits a faster degradation rate compared to PLGA 75 : 25 due to higher glycolic units. Similarly, PLGA 75 : 25 shows faster degradation than PLGA 85 : 15 [69]. Hence polymers with degradation rate varying from weeks to years can be fabricated by tuning lactide to glycolide units and lactide stereoisomeric composition [56]. Another factor that affects the degradation properties is the molecular weight. Since the polymer chain size is directly proportional to the molecular weight. Polymers with lower molecular weight will exhibit faster degradation rates because they have small polymer chains, which degrade much faster than long polymer chains [70]. Therefore the degradation and drug release rate can be customized to achieve controlled release over several weeks to months by varying polymer ratio and molecular weight. 

Another important factor is the selection of therapeutic/drug molecule to match the type of implantable delivery systems. Studies using biodegradable polymers have shown that the chemical properties of drug can affect the rate of polymer degradation, rate of water absorption into the matrix, and drug release rate [71, 72]. The potential formation of polymer-drug matrix could also affect (a) stability of drugs, (b) drug release pattern, (c) safety profiles of drug and polymers, and (d) pH and osmolality of ocular fluids. Since the goal of drug delivery in glaucoma management is to improve therapeutic efficacy while minimizing systemic and local toxicity; it is very important to optimize the process of drug loading and ocular release parameters to avoid dose dumping or erratic drug release profiles. The fact still remains that only within the therapeutic window will drugs maintain balance between efficacy and safety. Even at therapeutically effective and safe drug concentrations, prolonged exposure of ocular tissues to inserted implant might trigger inflammatory reactions to varying degrees in different patients.

In recent years, prostaglandin analogs (e.g., bimatoprost, latanoprost, and travoprost) are being considered over beta-blockers (e.g., timolol maleate) as effective topical agents for lowering IOP in glaucomatous conditions. The prostaglandin analogs are enzymatically cleaved and converted to their active form after they are delivered to the intended site [10]. Ocular implants for prodrug-based therapies should preserve the rate and extent of ocular activation to therapeutically active form of the drug. Currently a phase 1 efficacy, safety, and tolerability study of latanoprost sustained release insert is underway at the University of Kentucky [43].

### 3.2. Choice of Sterilization Process

All ocular implants for sustained drug release must be free from potential health hazards. As such, sterilization is required to destroy or eliminate unwanted living microorganism contamination prior to implantation. Sterilization can be carried out by a number of methods such as aseptic method/manufacture, gamma irradiation, heating, and gassing with ethylene oxide [73, 74]. It is known that sterilization methods could modify the polymer properties and impact drug loading and release profiles. For instance, heat sterilization could cause degradation of drug and alteration of polymer micro- and/or macroscopic mechanical properties, while autoclaving is not recommended since it can trigger drug loss or migration of drug to the outer surfaces of implants [75]. The choice of gamma irradiation should be based on the chemical stability of implants and loaded drug. Free radicals that are formed during gamma sterilization can initiate chemical modification of the materials used in polymeric matrix of implants. It was reported that POE III polymer degradation was induced by gamma irradiation [46]. The choice of a sterilization method should be done carefully to preserve the integrity of the implants as well as attain satisfactory sterility assurance level.

### 3.3. Level of Surgical Procedure Required for Implantation

A major challenge in ocular drug delivery to the posterior segment is the multiple layers of protective blood-ocular barriers that limit drug access to intraocular tissues [76]. As most vision impairing diseases are associated with the posterior eye segment, the administration of drug is becoming even more challenging [30]. The difficulty in obtaining effective therapeutic concentration of drugs using conventional methods has led to the exploration of numerous sustained-release glaucoma drug delivery systems. Some of these systems are still in the investigational phase, being tested in preclinical models and others are approaching clinical study. 

The level of surgical procedure involved in securing the implant at the intended site will play an important role in defining the safety and acceptability of the device. Intraocular methods such as intravitreal administration involving direct injection through the pars plana is the direct delivery route to posterior eye segment because it provides high drug concentrations at the vitreous and minimizes adverse systemic effects [57]. Due to the invasive nature of administration, it is important to develop implants using drug reservoir to provide extended drug delivery over long duration to minimize frequent dosage. Further, repeated administration via this route could lead to ocular complications such as retinal detachment, vitreous hemorrhage, irritation, and infection at the implantation site [9, 77]. Hence, even though intravitreal implants are effective for targeted therapy with increased ocular bioavailability, the invasive procedures that are required to secure the implants at the target site and the subsequent surgery to retrieve the device in the event of any complications will create major liabilities in clinical settings. 

The primary criterion of all is getting patients to tolerate the mode of administration of implantable delivery. Thus there is a growing need to investigate patient friendly delivery routes to eliminate discomfort and side effects resulting from the method of delivery to overcome the fundamental problem of patient adherence. More recently periocular pathways such as subconjunctival, peribulbar, retrobulbar, and subtenon routes are being considered for drug administration to the vitreous cavity by crossing the sclera, choroid, and RPE barriers [76, 78]. The usage of less invasive extraocular biodegradable systems such as episcleral, conjunctival, and subconjunctival implants can reduce potential complications associated with intravitreal delivery [4, 43, 79]. Periocular routes such as subconjunctival and subtenon administration are most widely studied due to their close proximity to the sclera [57]. After subconjunctival injection, the drug must penetrate across the sclera, which is highly permeable to large molecules than the cornea. Hence this route can be used to deliver large molecules such as proteins and peptides [80]. However, delivery to the retina is more complicated as the choroidal circulation and tight junctions of the RPE restrict penetration of the drug [81]. The elimination of administered drug via the choroid, a network of blood vessels between the sclera and retina, has an important role in periocular drug loss resulting in low bioavailability at the target tissue [57]. As periocular pathways hold good promise of accessing the retina and vitreous via less invasive methods, mechanisms that aid drug retention and ocular permeation should be considered. For instance, targeted delivery platforms that employ colloidal carriers such as micro/nanoparticles, niosomes, liposomes and microemulsions can enhance permeation across ocular barriers and prevent degradation and elimination [82, 83]. A thorough understanding of the drug clearance pathways in sclera, choroid and RPE should help develop new materials and injection techniques to achieve multifold tissue permeation to further improve dosing convenience and efficacy.

### 3.4. Size of Implants

The size of the implants will play substantial roles in the feasibility of securing implants with minimal invasiveness during regular doctor's visits. Biodegradable devices with large surface areas tend to degrade faster than those with small surface areas, which may be due to the actual area of the implant in contact with ocular fluids [29]. Reports from a study on PLGA-based device showed that degradation by bulk erosion (resulting in rapid drug release) was more pronounced with implants that have large surface area [84]. Further, larger solid implants can trigger foreign body reaction, consisting of fibroblasts, foreign body giant cells, and macrophages on the surface of the implant. As a consequence, the fibrous capsule formed around the implant prolongs its rate of degradation or elimination from the biological environment [61]. A major challenge in designing implants with small surface area is that the devices are usually loaded with large amounts of drug to achieve therapeutic efficacy over long time periods. Meanwhile, overloading of drug within the polymer matrix may lead to an undesirable initial burst, which is problematic [29]. Thus, strategies that will aid in achieving and retaining homogenous drug concentrations in the polymer matrices at a given time interval could ensure desirable drug release profiles. Generally, implants that are geometrically small would be well tolerated (no irritation) than larger implants, which could translate into improved patient acceptance and adherence to treatment [31].

### 3.5. Drug Release Kinetics

The ability of implants to continuously release drug over extended period of time is crucial especially for glaucoma that requires chronic drug administration. It is highly desirable to avoid erratic drug release with potential implications in therapeutic effectiveness and toxicity. Over-all, biodegradable systems are more prone to nonlinear drug release kinetics and increased burst effects compared to nonbiodegradable systems [29]. Also, burst release patterns are more pronounced with hydrophilic drugs in polymer matrices that are usually hydrophobic due to their poor drug-polymer interaction. Considering biodegradable systems, drug release pattern may follow three phases involving initial burst, diffusive release (regulated by polymer degradation rate, surface area, and solubility of loaded drug), and the final burst from disintegrating polymer matrices [46]. The solubility of the drug determines its loading capacity and the higher the solubility the more uniform the distribution of drug within the polymer matrix. Uniform drug distribution further reduces the risk of unwanted burst release [85, 86]. Overloading of drug and nonuniform distribution of drug within the polymer matrix can result in increased release during initial burst, which can cause undesirable ocular effects and inflammatory responses. The release profile of implantable delivery can be affected by the following: (1) amount of drug loaded, (2) surface area and volume of implant, (3) type of polymer and composition, (4) average molecular weight of polymer, and (5) solubility of the drug. 

**Figure 2:**
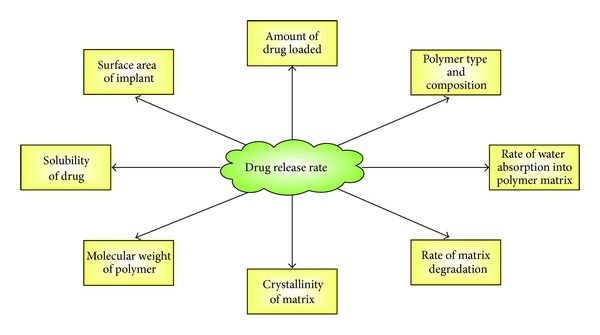
Schematic of the various factors that could affect drug release rate from ocular implants.

Continuous attempts are being made to minimize the burst effects and achieve linear drug release kinetics [28, 29]. Formulation strategies that can enhance drug dispersion in the polymer matrices using suitable drug carriers and emulsifying agents can stabilize the burst effect and result in a drug release rate that correlates with polymer degradation. Also in order to maintain constant release of drug, it is important to use geometrical shapes that will minimize reduction of surface area with degradation [54]. The various factors that affect drug release rate from ocular implants are summarized in Figure 2. 

A viable approach to achieving desired drug release profiles is by modifying the polymer composition. For instance, some studies have demonstrated that combining two PLA monomers of high and low molecular weights resulted in biphasic release pattern (eliminating the final burst) and achieved pseudozero order kinetics comparable to nonbiodegradable systems [29, 48, 87]. In these implants, the high molecular weight polymer provided the framework and restricted the degradation rate of low molecular weight polymer and the low molecular weight polymer gradually degraded releasing drug in a controlled manner [28]. Further, by changing the blend ratio of high and low molecular PLA, the duration of drug release can be controlled [48]. The molecular weight is directly related to the rate of biodegradation, and thus the greater the molecular weight the slower the speed of degradation, and rate of drug release is also modulated. From the experimental results, it can be suggested that blended polymer matrices could offer promising avenues for sustained intraocular drug delivery over few months to a year. Additionally, the choice of polymer matrices must be determined carefully based on the physiochemical properties of the drug to be loaded and the expected duration of release.

### 3.6. Perspectives on Future Glaucoma Implantable Drug Delivery Systems

The classification of glaucoma as a neurodegenerative disease has presented the urgent need to develop strategies for drug delivery to the posterior segment. In a recent glaucoma clinical trial design and endpoints symposium, FDA emphasized the importance of structural parameters that involve optic disc and retinal nerve fiber layer (RNFL) changes in assessing clinical outcome of new glaucoma therapies [88]. Since preserving vision is the primary goal in glaucoma treatment, current knowledge of the pathologic factors resulting in optic nerve damage with or without associated elevated IOP is limited. Considering that elevated IOP is a major risk factor in glaucoma, therapeutic interventions on lowering IOPs alone could help in managing the progression of the disease but may not address the underlying vulnerability of RGCs to degeneration [13]. The key issue is that the current functional measures (i.e., visual acuity, visual field, and contrast sensitivity) used for evaluation of new drugs and devices do not provide a meaningful relationship between visual field loss and structural change in optic nerve [89]. Even though there are many clinically available implantable delivery systems for ocular diseases and disorders, there is none approved (at the time of writing) for glaucoma. To set a stage for new treatments in the future, FDA expressed openness to use structural metrics to measure progression of the disease if they (1) demonstrate strong correlation to clinically relevant functional changes, (2) provide reproducible measures of clinically significant changes, and (3) are beneficial to the patients [88]. With improvements in imaging technologies, it is expected that combining structural and functional measures can surmount some of the issues in glaucoma clinical trial design and move therapies forward for FDA approval. As such, we consider that effective delivery strategies should implement combination therapeutics that will address the currently identified pathological factors involved in glaucoma. Such an approach will incorporate therapeutic agents that target lowering IOP as well as neuroprotective agents directed at preserving RGC degeneration and apoptosis. In this regard, implantable delivery systems in glaucoma could offer many advantages such as (1) reducing frequency of dosing through sustained drug delivery in order to ensure patient adherence; (2) improving drug delivery to the posterior segment thereby enhancing treatment outcomes.

Considering that cases of glaucoma are estimated to increase in the coming years, it is important to tackle the challenges of drug delivery to the eye, as it is a complex organ that is difficult to access both topically and systemically. The fact is that most patients and clinicians will prefer less invasive methods of securing implantable delivery systems in the eye. We strongly believe that there are many factors to consider such as (i) placement of implants should be convenient and ensure less frequent drug administration; (ii) patients should be able tolerate implant placement; (iii) biomaterials used in implant preparation as well as byproducts from possible implant degradation should be safe, biocompatible, and easily eliminated; (iv) ocular drug release from implant should be predictable while avoiding the dangers of burst drug releases and dose dumping; and (v) implantable delivery systems should not compound patients medical conditions through elevation of IOP, interference with vision, and triggering inflammatory responses. We considered that a worthwhile approach of addressing these issues with predictable drug release profiles from implantable delivery systems might involve the application of stimuli-responsive (smart) strategies. Ocular implants that employ smart delivery systems can potentially offer great benefits over traditional systems since release of therapeutic agents can be controlled based on disease-specific (proximal) or nondisease-specific (external) stimuli [90]. It is envisaged that current advancement in the area of stimuli responsive polymers can open up new avenues for the development of novel implantable delivery systems and formulations for the treatment of glaucoma with clear and compelling long-term benefits. 

## 4. Conclusion

Glaucoma is a group of multifactorial neurodegenerative diseases that collectively are the leading cause of irreversible blindness worldwide. The incidence is expected to increase remarkably in the next decade based on estimated growing aging population. Development of effective sustained intraocular drug delivery systems is a major unmet need in glaucoma management. The paper critically evaluated the rationale for implantable delivery systems as strategies of relieving the burden of protracted drug administration while maintaining high intraocular drug bioavailability. Major challenges of glaucoma-focused implantable ocular drug delivery were discussed while offering possible strategies on achieving and sustaining (i) therapeutic efficacy, (ii) desired therapeutic outcomes, and (iii) patient adherence and acceptance. It is considered that recent progress in the field of stimuli-responsive biomaterials could hold great promise in sustained drug delivery in glaucoma.

## Figures and Tables

**Table 1:** Examples of sustained release delivery systems studied in glaucoma-induced preclinical models.

Implant type	Materials	Drug loaded	Duration of drug release	Delivery method	Reference
Biodegradable PLGA film	PLGA 50 : 50	Ethacrynic acid (ECA)	10 days	Implanted in sclera	[32]
Biodegradable PCL + Lutrol F 127 disk	PCL + Lutrol F 127	Trusopt (Dorzolamide Hydrochloride)	6 months–1 year	Subconjunctival implantation	[33]
Microspheres	50 : 50 blend of PLGA 502H + PLA	Timolol Maleate	3-4 months	Subconjunctival injection	[34]
Liposomes	Dipalmitoylphosphatidylcholine (DPPC) lipids	Latanoprost	2-3 months	Subconjunctival injection	[35]

**Table 2:** Examples of sustained release delivery systems for glaucoma that are under clinical development.

Drug	Implant type	Administration route	Manufacturer	Status	Information source
Bimatoprost	Sustained release punctal plug	Inserted into lid puncta	Vistakon Pharmaceuticals	Completed	[36, 37]
Latanoprost	Punctal plug	Inserted into lid puncta	QLT	Completed	[38]
Latanoprost	Biodegradable DURASERT (Latanoprost)	Subconjunctival implantation	pSivida Corp.	Ongoing	[39]
Latanoprost	Nonbiodegradable pellets of Latanoprost coated with EVA	Subconjunctival implantation	Aerie Pharmaceuticals	Ongoing	[40]
Travoprost	Sustained release travoprost punctum plug	Inserted into canaliculus of the eyelid	Ocular Therapeutix	Recruiting	[41]
Ciliary neurotrophic factor	Nonbiodegradable NT 501	Pars plana implantation	Neurotech	Ongoing	[42]
Latanoprost	Biodegradable slow release (SR) insert	Subconjunctival implantation	Sunil Deokule & Pfizer	Recruiting	[43]
